# A rare case of herniated duplex collecting system causing obstructive uropathy

**DOI:** 10.1186/s12894-020-00652-z

**Published:** 2020-07-01

**Authors:** Christopher El Mouhayyar, Haoyang Wang, Laith Hattar, Fang-Yu Liu, Karen Feghali, Vaidyanathapuram Balakrishnan

**Affiliations:** 1 Department of Medicine, St. Elizabeth’s Medical Center, Boston, MA USA; 2 Department of Medicine, Tufts University School of Medicine, Boston, MA USA

**Keywords:** Uretero-inguinal hernia, Duplex collecting system, Acute kidney injury

## Abstract

**Background:**

An inguinal hernia is the protrusion of intraabdominal organs through an opening in the abdominal wall. Structures such as small and large intestines are commonly contained within inguinal hernias. However, uretero-inguinal hernia of the native collecting system is an extremely rarely reported entity. If unrecognized, acute kidney injury due to obstructive uropathy or serious intraprocedural ureteral injuries during hernia repair can occur. A duplex collecting system is a congenital kidney anomaly with an incidence of 0.8%. A uretero-inguinal hernia involving duplicated ureters has not been previously described in literature. Here we report a case of obstructive uropathy secondary to uretero-inguinal hernia involving duplicated ureters.

**Case presentation:**

A 78-year-old male known to have a left sided inguinal hernia presented to the Emergency department with two weeks of intermittent suprapubic tenderness, dysuria, frequency, urgency, frothy urine as well as nausea and vomiting. Workup on admission revealed an elevated creatinine of 2.8 mg/dl. CT imaging revealed duplicated left sided ureters with left inguinal hernia containing the ureters. There was cystic ureteral dilation within the herniation sac as well as moderate left hydroureteronephrosis. Patient had an elective inguinal hernia repair with left ureteral stent placement. Following the surgery, he had recovery of kidney function to the previous baseline serum creatinine of 1.5 mg/dl.

**Conclusion:**

A duplex collecting system arises when two ureteral buds are formed during fetal development. However, diagnosis can be made in rare instances during adulthood when duplex collecting systems are usually found incidentally. Uretero-inguinal hernias have been reported as a common complication of renal transplant. However, uretero-inguinal hernias in native kidneys are considered an uncommon finding, especially with a duplex collecting system. When patients present with herniation and acute kidney injury, it is important to rule out the possibility of uretero-inguinal hernia to minimize complications such as obstructive uropathy and kidney failure. CT scan providing cross-sectional imaging is the ideal modality for identification of the site and etiology of urinary tract obstruction and site of herniation. If during imaging, an obstructive uropathy is observed, a nephroureteral stent or nephrostomy tube can be inserted to protect the ureter as well as relieve the obstruction, respectively.

## Background

An inguinal hernia is the protrusion of intraabdominal or extraperitoneal organs through an opening in the abdominal wall that is widely prevalent amongst male population [1, 2]. A wide range of structures such as small bowel, large bowel and bladder are commonly contained within inguinal hernias. However, uretero-inguinal hernia is a rarely reported entity that is more frequently seen following a kidney transplant. Even further, uretero-inguinal hernias of the native kidneys have rarely been reported. This phenomenon is potentially associated with acute kidney injury due to obstructive uropathy or serious ureteral injuries if unrecognized prior to surgical hernia repair. A duplex collecting system is a common congenital kidney anomaly, with an incidence of 0.8% [3]. A uretero-inguinal hernia involving duplicated ureters has not been previously described in literature (Table 1). Here we report a case of obstructive uropathy secondary to uretero-inguinal hernia involving duplicated ureters.

**Table 1:** Table showing Case reports in literature describing ureteroinguinal hernia in transplant vs native kidneys with a single or double collecting system, findings on imaging as well as the modality of management

Authors	Collecting System	Kidney	Imaging Finding	Management
He et al. [4]	Single	Native	Left Hydrourertero-nephrosis	Nephro-ureteral stent
Hong et al. [5]	Single	Native	Left Hydrourertero-nephrosis	Conservative
Lu et al. [6]	Single	Native	Right Hydrourertero-nephrosis	Conservative
Eilber et al. [7]	Single	Native	Right Hydrourertero-nephrosis	Conservative
Sidqi et al. [8]	Single	Native	Left Uretero-nephrosis	Lichtenstein repair
Yahya et al. [9]	Single	Native	Right Hydrourertero-nephrosis	Lichtenstein repair Nephro-ureteral stent
Otani et al. [10]	Single	Transplant	Right hydronephrosis	Nephro-ureteral stent
Furtado et al. [11]	Single	Transplant	Left Hydrourertero-nephrosis	Hernia Repair
Weingarten et al. [12]	Single	Transplant	Left Hydronephrosis	Nephro-ureteral catheter Hernia Repair
Osman et al. [13]	Single	Transplant	Right Hydrourertero-nephrosis	Hernioplasty Nephrostomy tube
Abu-areda et al. [14]	Single	Transplant	Right Hydrourertero-nephrosis	Nephrostomy tube
Won et al. [15]	Single	Native	Pelvic–ureteric junction obstruction	Conservative
Malde et al. [16]	Single	Native	Left Hydrourertero-nephrosis	Mesh Hernia Repair
Sanchez et al. [17]	Single	Transplant	Right Hydrourertero-nephrosis	Hernioplasty

## Case presentation

**Fig. 1:**
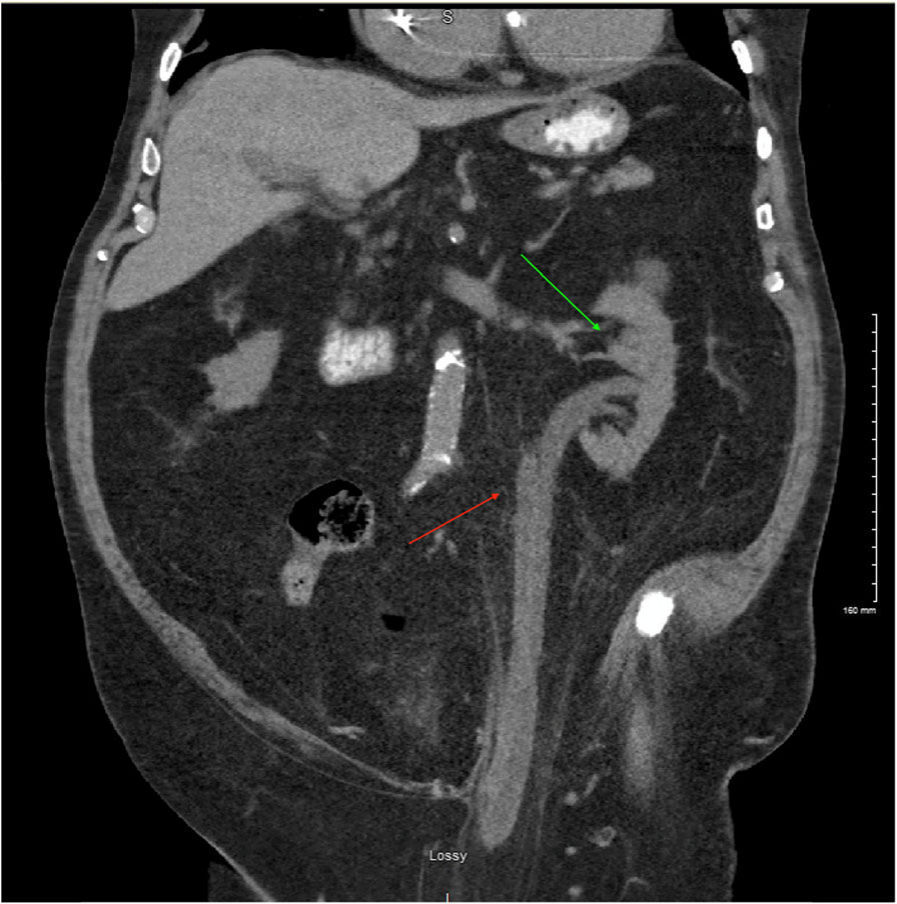
CT scan of the abdomen and pelvis showing the hydroureternephrosis. (Green arrow: Hydronephrosis, Red arrow: ureteronephrosis)

**Fig. 2:**
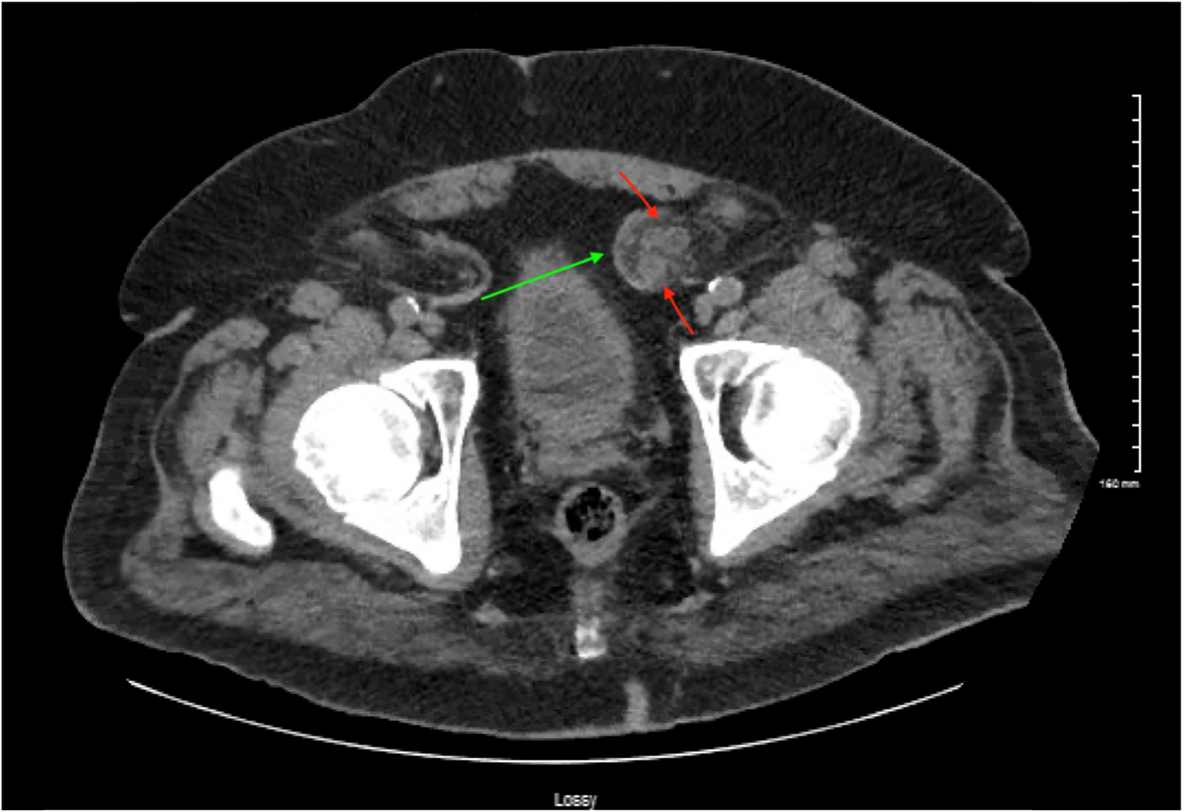
CT scan of the abdomen and pelvis showing the uretero-inguinal hernia involving duplicated ureters (Green arrow: Hernia, Red arrow: Two ureters)

A 78-year-old male with past medical history significant for CKD stage 3 known to have a left sided inguinal hernia presented to the Emergency department with two weeks of intermittent suprapubic tenderness, dysuria, frequency, urgency, frothy urine as well as nausea and vomiting. Workup on admission revealed an elevated creatinine of 2.8 mg/dl from a baseline of 1.5 mg/dl, with leukocytosis of 7000. Urine analysis was performed that revealed large leukocyte esterase, urine WBC greater 180, and white blood cell clumps. The patient was diagnosed with acute kidney injury as well as a urinary tract infection. Computed tomography of the abdomen and pelvis revealed duplicated left sided pelvis and ureters with the left inguinal hernia containing the ureters with cystic ureteral dilation within the herniation sac as well as moderate left hydroureteronephrosis (Figs. 1 and 2). The patient was managed with volume repletion and antibiotics for his urinary tract infection. There was some improvement in kidney function although not to his previous baseline. He was discharged with an outpatient urology follow up and was subsequently readmitted for an elective paraperitoneal inguinal hernia repair with a Kumpe catheter placement up to the left kidney externalizing through the urethral meatus.

When it comes to the inguinal hernia repair, a large portion of the herniated extraperitoneal fat was freed from its incarcerated position in the scrotum and then dissected back to the internal ring. A typical mesh repair was not done in order to avoid placing the mesh in direct contact with the ureter causing erosions as well as ureteral structuring further down the line. Thus, the internal ring was primarily closed first to maintain the ureter in a reduced position deep to the ring then a Lichtenstein repair was done using a polypropelene patch that was sutured to the tissues overlying the pubic tubercle medially to the shelving edge of the inguinal ligament inferiorly and to the conjoined tendon superiorly, laterally the arms of the patch were passed around the spermatic cord at the internal ring and sutured together and to the tissue lateral to the internal ring.

**Fig. 3:**
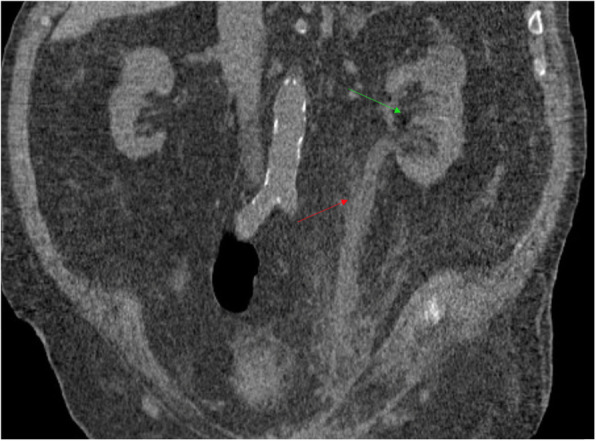
CT scan of the abdomen and pelvis post-operation day 20 showing improvement in hydroureternephrosis. (Green arrow: Hydronephrosis, Red arrow: ureteronephrosis)

Following the surgery, he had recovery of kidney function to the previous baseline serum creatinine of 1.5 mg/dl. (Table 2) (Fig. 3).

**Table 2:** Table showing Creatinine (Cr: mg/dL) as an indication of kidney function over time (prior to the operation and post operation) POD: post-operative day. PrOD: Pre-operative day

Days	PrOD	POD1	POD2	POD3	POD4	POD5
Cr (mg/dL)	2.8	2.2	1.75	1.65	1.5	1.43

## Discussion and conclusion

Ureteral development begins in fetus at the age of four weeks with the ureteral bud branching from the mesonephric duct [18]. The ureteric bud is responsible for the formation of the collecting system. If two ureteral buds arise, the caudal ureter drains the lower pole and the cephalic ureter drains the upper pole [19]. Patients usually present in childhood with recurrent UTIs, flank pain, incontinence and hematuria [20]. However, diagnosis can be made in rare instances during adulthood when duplex collecting systems are usually found incidentally on abdominal imaging or during surgery [21–23].

Uretral herniation can occur at different sites including inguinal, femoral, sciatic, thoracic and parailiac [24, 25]. Uretero-inguinal hernias have been reported as a common complication of renal transplant [26].However, uretero-inguinal hernias in native kidneys are considered an uncommon finding. They are divided into two types: the paraperitoneal or extraperitoneal with paraperitoneal accounting for majority of cases [6, 27].

A paraperitoneal hernia occurs when the ureter is adherent to the posterior peritoneum and is herniated alongside the peritoneal sac into the inguinal canal. It is a sliding type of hernia thought to be caused by traction of the underlying structures or adhesions that attach the ureter to the posterior peritoneum. This type of herniation is often accompanied by herniation of other organs such as the colon [15].

On the other hand, extraperitoneal herniation is herniation of the ureter without the peritoneal sac. It is theorized to be due to a congenital embryonic defect that resulted in the fusion of the ureter and the genitoinguinal ligament due to the failure of separation of the ureteric bud from the wolffian duct [26, 28]. However, one case report attributed the formation of extraperitoneal ureteral hernia to adhesions from a previous hernia repair [29].

Uretero-inguinal hernias can potentially cause obstructive uropathy and in some cases lead to kidney failure if left undiagnosed. Further, if undiagnosed, iatrogenic ureteral injury can occur during hernia repair [30].

Obesity as well as deficiency in collagen synthesis are risk factors for ureteral hernias. These hernias are more frequent on the right side compared to the left due to differences in fascia of Toldt morphology [30].

Ultrasound is the ideal first line imaging modality to assess the upper urinary tract as it can identify hydronephrosis as well as the integrity of the proximal ureter. However, ultrasound of the urinary tract does not routinely include assessment of the inguinal orifices making the tracking of the ureter in the retroperitoneal space a challenge. Cross-sectional imaging (CT scan or MRI) are more reliable for identification of the site and etiology of urinary tract obstruction and can allow identification of the site of herniation as well as anatomical classification with CT remaining more easily accessible between the two modalities.

Herniorrhaphy is considered the treatment modality for uretero-inguinal hernias causing obstructive symptoms [7]. If during imaging, an obstructive uropathy is observed, a nephroureteral stent or nephrostomy tube can be inserted to protect the ureter as well as relieve the obstruction, respectively [4].

A uretero-inguinal hernia is a rare condition that can potentially lead to obstructive uropathy. CT is currently the modality of choice for identification, diagnosis as well as anatomical classification of the hernia and its possible complications. In this case report, we describe a unique uretero-inguinal hernia involving a duplicated ureter causing obstructive uropathy. Recognizing such an entity is important in identifying causes of acute kidney injury and preventing surgical complications.

## Data Availability

Data sharing is not applicable to this article, as no datasets were generated or analyzed for the purpose of the following study.

## Abbreviations

CT scan: Computerized Tomography scan
MRI: Magnetic Resonance Imaging
POD: post-operative day
PrOD: Pre-operative day
Cr: Creatinine
